# The Association Between Internet Game Addiction and Social Anxiety Symptoms Among Adolescents in The Kingdom of Saudi Arabia

**DOI:** 10.1192/j.eurpsy.2025.838

**Published:** 2025-08-26

**Authors:** B. A. Alsubaie, N. Alhujaili, S. Alzahrani, F. Aljahdali, W. Alghamdi, M. Almatrafi, A. Alghamdi

**Affiliations:** 1 Psychiatry; 2 Medicine, King Abdulaziz University, Jeddah; 3 Psychiatry, King Salman Specialist Hospital, Hail, Saudi Arabia

## Abstract

**Introduction:**

Internet gaming addiction (IGA) can have a significant impact on the characteristics of individual gamers, and may even be a contributing factor to the development of social anxiety symptoms. This study of adolescent Saudi Arabian Internet gamers examined the influences of Internet gaming time and probable Internet Gaming Disorders (IGDs). It investigated the association between Internet game addiction and social anxiety symptoms among them.

**Objectives:**

The aim of this study was to investigate the association between internet game addiction and social anxiety symptoms among Saudi adolescents. Since very few researches examined the association between internet game addiction and psychiatric illnesses among Saudi youth, Our study focused on social anxiety symptoms which has not been done so far.

**Methods:**

A cross-sectional, survey was conducted from March to June of 2023 among adolescents in the Saudi population. Probable Internet Game Addiction was measured by using the DSM-5 checklist and the Arabic version of the Social Phobia Inventory (SPIN) was used to evaluate social phobia or anxiety.

**Results:**

A total of 764 participants were enrolled in the current study, majority of gaming addiction participants were 15-16 years old. There was a significant association between gender and gaming addiction (p-value ≤ 0.05). 86.6% of Saudis have been significantly associated with gaming addiction. The majority of the participants have moderate social phobia compared to severe social phobia. There was a significant association between social phobia and age (p-value ≤ 0.05). 34.9% of male participants have moderate social phobia and 24.6% of females have moderate social phobia. There was an insignificant association between social phobia and nationality. The participants with gaming addiction showed 37.2% had mild, 60.5% had moderate, 68.1% had severe and 79.5% had very severe social phobia. There was a significant association between Social Phobia and gaming Addiction (p-value≤0.05).

**Conclusions:**

This study examines the association between video game addiction and social anxiety amongadolescents in Saudi Arabia. Despite its limitations of less sample size and small setting, the findings imply a significant correlation between IGA and these psychological concerns, particularly among male adolescents. Excessive video gaming use leads to higher levels of social anxiety. Future research should adopt experimental or longitudinal designs to establish causal relationships and consider the potential reciprocal nature of the association. Mental health educators and practitioners should be mindful of the adverse impacts of excessive video gaming, with a specific focus on male adolescents who might be more susceptible to heightened social anxiety.

**Disclosure of Interest:**

B. Alsubaie Employee of: General physician in psychiatry department. Declare no conflict of interest, N. Alhujaili Consultant of: Psychiatry. Declare no conflict of Interest, S. Alzahrani Employee of: General physician. Declare no conflict of interest, F. Aljahdali Employee of: Medical Intern. Declare no conflict of interest, W. Alghamdi Consultant of: Associate professor of psychiatry. Declare no conflict of interest, M. Almatrafi Consultant of: Psychiatry. Declare no conflict of Interest, A. Alghamdi Consultant of: Psychiatry. Declare no conflict of interest

